# Supplementary material for: Analysis of chi angle distributions in free amino acids via multiplet fitting of proton scalar couplings
Source: Magn Reson (Gott). 2024 Aug 19;5(2):103–20. doi: 10.5194/mr-5-103-2024 (PMC11570886; doi:10.5194/mr-5-103-2024)
Supplement: The supplement related to this article is available online at: https://doi.org/10.5194/mr-5-103-2024-supplement. [file mr-5-103-2024-supplement.zip › Syed2024/data/fit1d_fitnmr_output.tar.gz!/fit1d/val/500/bmse000052_simulation_1_nmredata/1H/resonances_1d.pdf]

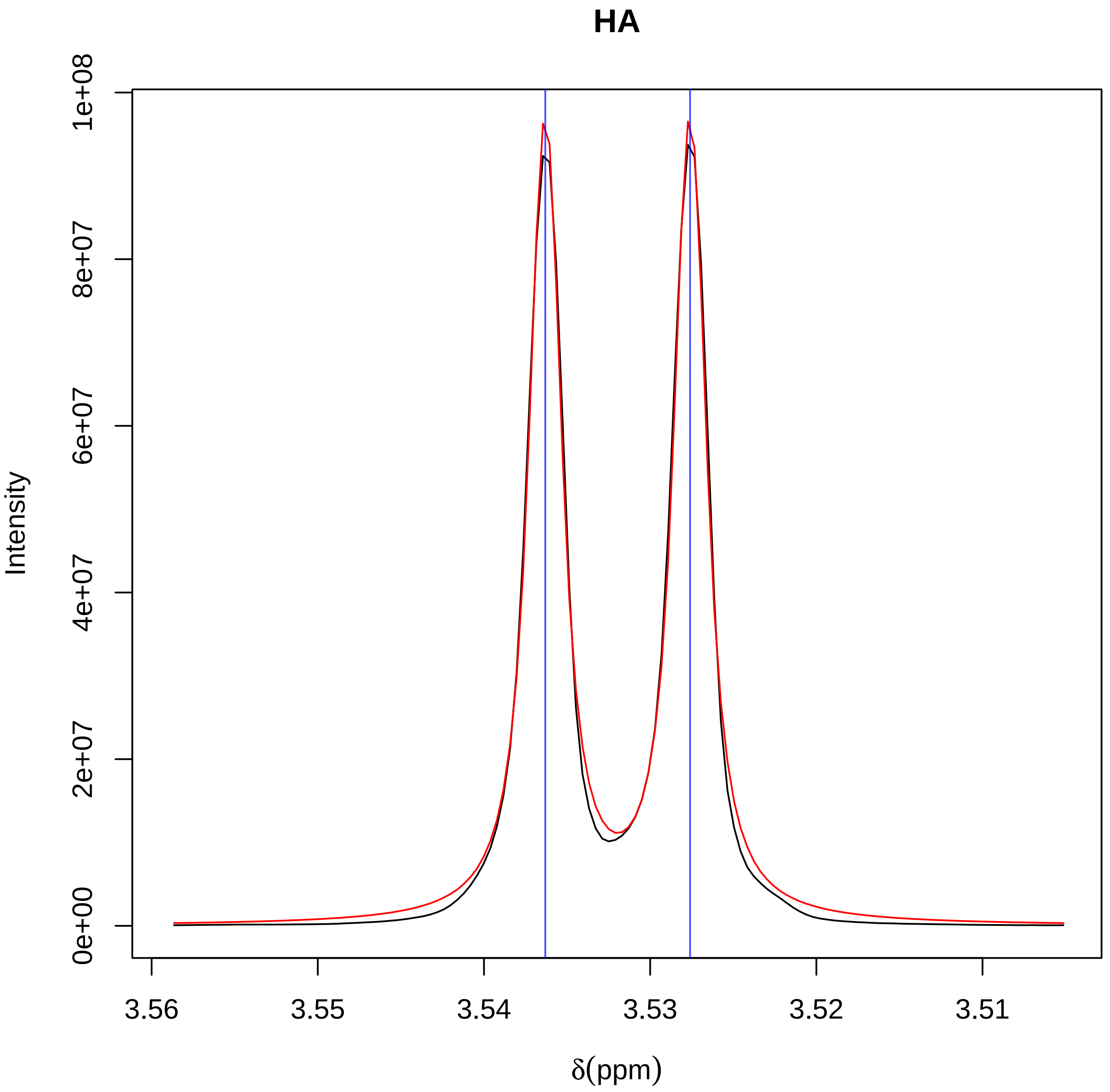
HA
Intensity
0e+00
2e+07
4e+07
6e+07
8e+07
1e+08
3.56
3.55
3.54
3.53
3.52
3.51
δ(ppm)

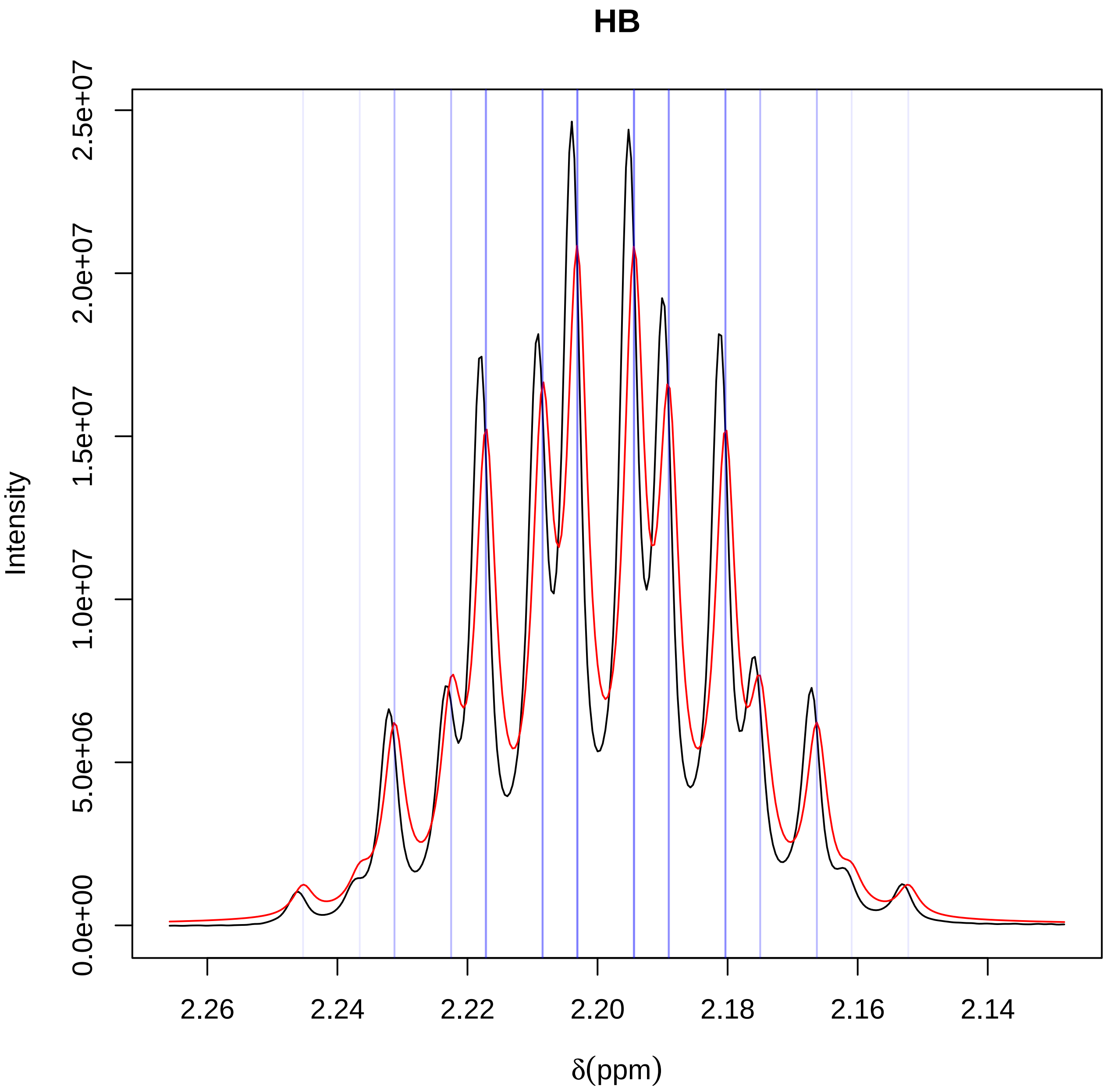

HB
Intensity
0.0e+00
5.0e+06
1.0e+07
1.5e+07
2.0e+07
2.5e+07
2.26
2.24
2.22
2.20
2.18
2.16
2.14
δ(ppm)

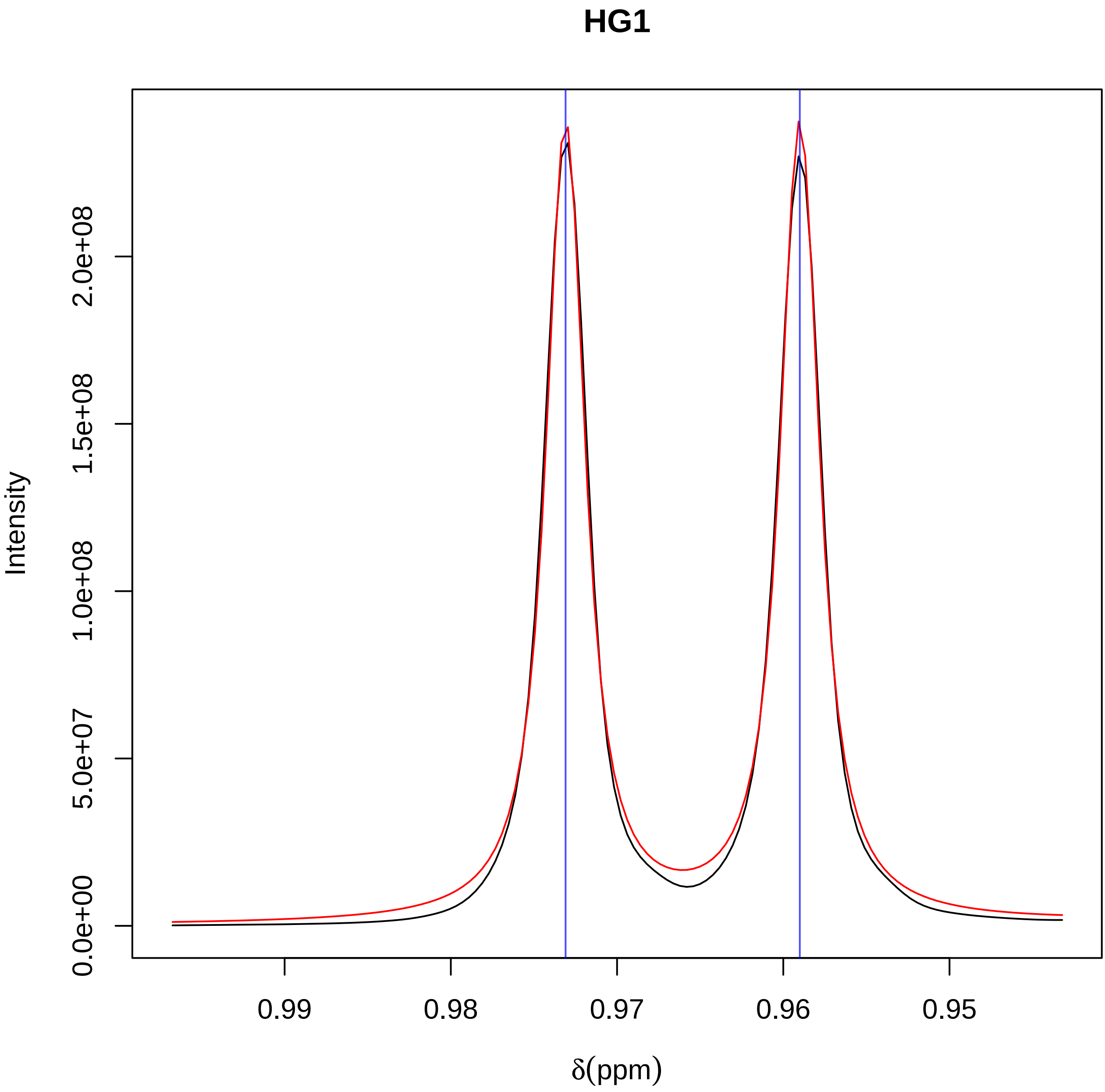

HG1
Intensity
0.0e+00
5.0e+07
1.0e+08
1.5e+08
2.0e+08
0.99
0.98
0.97
0.96
0.95
δ(ppm)

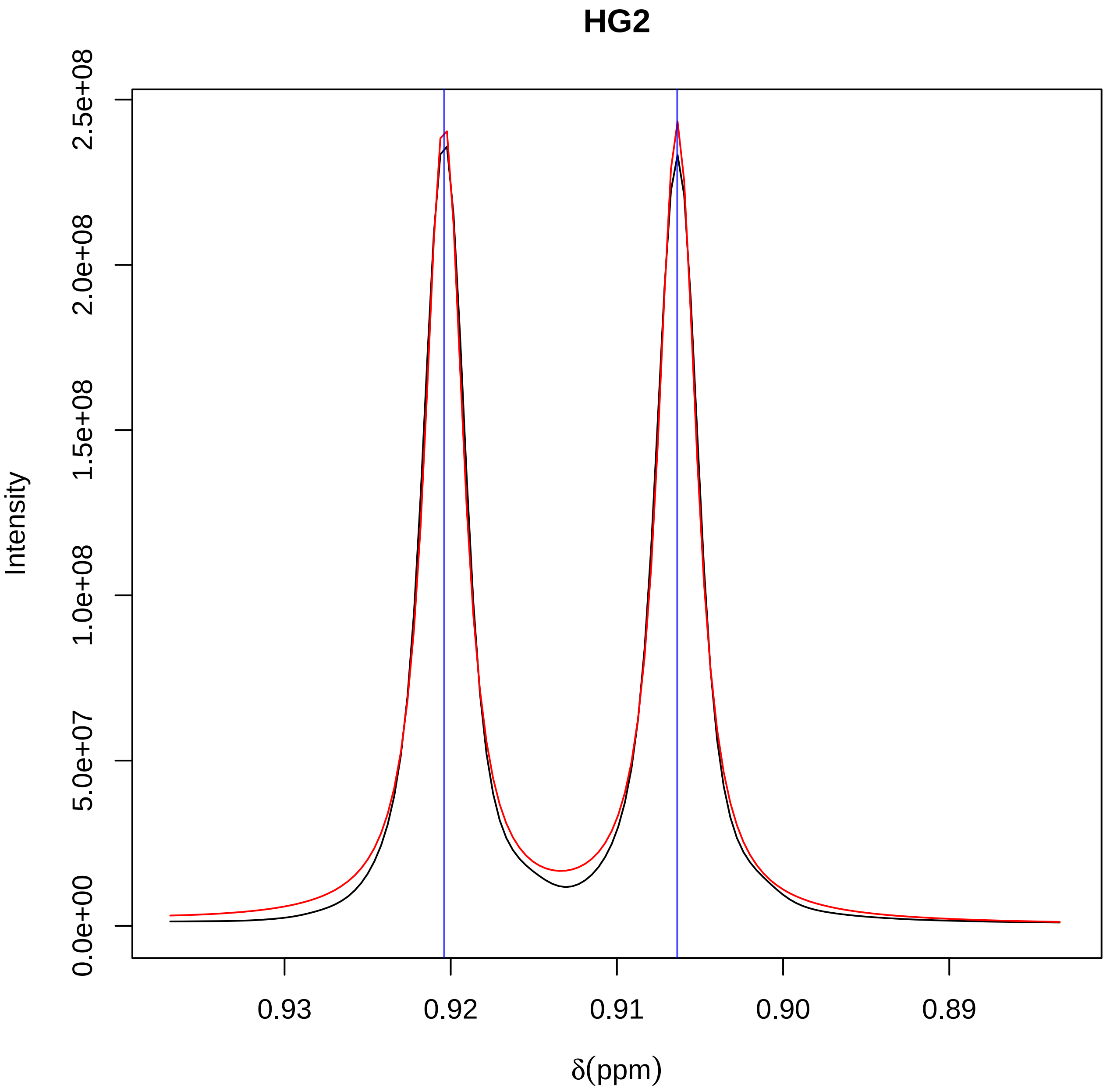

HG2
Intensity
0.0e+00
5.0e+07
1.0e+08
1.5e+08
2.0e+08
2.5e+08
0.93
0.92
0.91
0.90
0.89
δ(ppm)
